# Highly Automated Formal Proofs over Memory Usage of Assembly Code

**DOI:** 10.1007/978-3-030-45237-7_6

**Published:** 2020-03-13

**Authors:** Freek Verbeek, Joshua A. Bockenek, Binoy Ravindran

**Affiliations:** 8 Johannes Kepler University, Linz, Austria; 9 University of Birmingham, Birmingham, UK; 10 Virginia Tech, Blacksburg, VA USA; 11 Open University of The Netherlands, Heerlen, The Netherlands

**Keywords:** Formal Verification, Assembly, x86-64, Memory Usage

## Abstract

We present a methodology for generating a characterization of the memory used by an assembly program, as well as a formal proof that the assembly is bounded to the generated memory regions. A formal proof of memory usage is required for compositional reasoning over assembly programs. Moreover, it can be used to prove low-level security properties, such as integrity of the return address of a function. Our verification method is based on interactive theorem proving, but provides automation by generating pre- and postconditions, invariants, control-flow, and assumptions on memory layout. As a case study, three binaries of the Xen hypervisor are disassembled. These binaries are the result of a complex build-chain compiling production code, and contain various complex and nested loops, large and compound data structures, and functions with over 100 basic blocks. The methodology has been successfully applied to 251 functions, covering 12,252 assembly instructions.

## 1 Introduction

This paper presents a formal methodology for reasoning over the *memory usage* of functions in a software suite. Various security properties require knowledge on memory usage. For example, proving absence of buffer overflows requires proving that a function does not write outside certain memory regions. Control-flow integrity requires showing, among other things, that the return address cannot be overwritten [61]. The security property called non-interference requires reasoning over which parts of the memory are used by which functions [50].

Moreover, memory usage is crucial for *compositional* reasoning over assembly code. Typically, compositional reasoning requires proving that certain code fragments are spatially independent [45,47]. A proof of memory usage can be used to prove such independence, thereby allowing composition. Consider a function $g$ that at some point calls function $f$. Compositional reasoning means that a verification effort over $f$ can be reused for verification of $g$ without unfolding it. This *at least* requires that the verification effort over $f$ establishes that $f$ does not modify the stack frame of $g$. More generally, compositional reasoning requires

*at least* knowing that $f$ restricts itself to certain parts of the memory. This is exactly what is established by proving memory usage.

Memory usage cannot satisfactorily be expressed at the source-code level. As an illustration, consider formulating a property that a function cannot overwrite its own return address. This requires knowledge on the values of the stack and frame pointers, making it an *assembly-level* property. At the assembly level, one can easily express a property formulating that the memory at the top of the stack frame (where the return address is stored) should remain unmodified.

Reasoning over assembly, however, is complicated due to the semantical gap between assembly and source code. In assembly code, ostensibly simple computations can be implemented using complex sequences of low-level operations. For example, a simple integer division by 10 can be implemented with a series of bit-level operations. Assembly code does not have types. It is common to, e.g., mix logical bitwise operators with signed integer arithmetic, or floating-point operations with bitvector operations. Assembly code does not have a clear distinction between stack frame and heap. Whether some address refers to a local variable stored in the stack, a global variable, or part of the heap, is provable only by adding assumptions on memory layout. Finally, assembly does not have a clear notion of scoping. Function calls are not necessarily clearly delineated, and instead of assuming that a function cannot write to a variable it has no access to (such as a local variable of another function), this has to be proven.

The contribution of this paper consists of a formal, compositional and highly automated methodology for reasoning over memory usage at the assembly-level.[3] Our approach first uses untrusted tools to generate a *formal memory usage certificate* (see Section 2). This certificate contains 1.) theorems on memory usage, 2.) the preconditions under which memory usage can be shown, and 3.) *proof ingredients*. These proof ingredients contain assumptions on memory layout, control-flow information, and invariants. Section 2 provides an example of a function that theoretically can overwrite its own return address. We show that the certificate provides preconditions and a formal proof that a return-address-based exploit is not possible under those preconditions.

The certificate and the original assembly are loaded into an interactive theorem prover (ITP). Memory usage in general is an undecidable property (Rice's theorem [48]), which is why we aim for an ITP environment to allow user interaction when necessary. Using the proof ingredients, the certificate is formally proven correct with minimal user interaction, making use of customized proof strategies. Section 3 describes certificate verification and composition.

To demonstrate applicability and scalability, we apply the methodology to x86-64 binaries of the Xen hypervisor [13] (see Section 4). The binaries are obtained via the standard Xen build process, including optimizations. The binaries are decompiled to assembly using off-the-shelf disassembly tools. Our methodology is applied to 251 functions; for each function a certificate is automatically generated, and a proof is finished in the Isabelle/HOL theorem prover [44]. With-

[3] All code and proofs are publicly available [57].

out exception, the manual interaction consists of elementary interactive theorem proving such as applying the proper proof method.

While past work [38,41,25] on assembly-level formal verification exists, the degree of either scalability or automation is limited. As example of interactive theorem proving, Boyer and Yu verified machine-code implementations of various standard sort- and string functions, requiring over 19,000 lines of manually written proof code for the verification of roughly 900 instructions [8]. As example of automated theorem proving, Tan et al. presented an approach which takes about 6 hours for a 533-instruction string search algorithm [56]. In constrast, this paper involves a degree of user interaction of ≈85 lines of proof code per 1,000 lines of assembly. Our work is able to almost fully automatically verify 12,252 instructions from real world industrial binaries compiled by a real world build process. Section 5 discusses prior art, its contrast with the paper's work, and the paper's contributions. To the best of our knowledge, there is no related work that is able to achieve similar scalablity and automation on real world binaries.

## 2 Formal Memory Usage Certificates

Figure 1 provides an example of a formal memory usage certificate (FMUC). The FMUC is generated automatically from an assembly file. This assembly file may be produced from a binary using a disassembler such as `objdump`, IDA,[4] Ghidra's decompiler,[5] or Capstone [46]. In case source code is available, the assembly code can also be produced directly by a compiler. In this example, the C code of Figure 1a is used solely for presentation, the input to the FMUC generation is the assembly created by decompiling the corresponding binary. For each function in the assembly file, an FMUC is produced. External functions, for example due to dynamic linking, are treated as black boxes (see Section 3.4).

An FMUC consists of two parts: a *memory usage theorem* and its proof (see Figure 1c). The theorem consists of assumptions implying a Hoare triple [28,40] over the function. The Hoare triple is specific to memory usage. Intuitively, it means that from a state satisfying precondition $P$, after execution of code fragment $f$, the state satisfies postcondition $Q$ (as in normal Hoare triples). The Hoare triple also contains a *memory region set* $M$. Besides its regular meaning, the Hoare triple expresses that any write that occurs during execution of $f$ occurs within one of the memory regions in this set.

The term memory usage formally denotes an overapproximation of the memory written to by a function. Thus, any address that is not enclosed in one of the regions of $M$, is guaranteed to be preserved. Set $M$, however, will also include the memory regions read by the function, for verification purposes.

The precondition $P$ expresses that the instruction pointer `rip` is at the entry point of the function. It also provides initial symbolic values for all registers and memory regions that are read (e.g.,: $\texttt{rsp} = \texttt{rsp}_0$). Finally, it formulates that the return address is stored at the top of the stack frame. The postcondition $Q$

[4] https://www.hex-rays.com/products/ida/index.shtml

[5] https://ghidra-sre.org/

```
int main(int argc, char* argv[]) {
  int* a = (int*)argv;
  int* b = (int*)(argv + 4);
  *(int*)(argv + 2) = *a + *b;
  *(char*)argv = 'a'; int array[argc]; for (int i = 0; i < argc; i++) {
    array[i] = argv[i][0] * 2;
  }

if (is_even(argc)) {
    return array[argc];
  }
  return array[0];
}
```

(a) C Code

```
Block 1149->120b;
Loop
  Block 123e->1244;
  If SF ≠ OF Then
    Block 120d->123a
  Else Break Fi
Pool;
Block 1246->1249;
Block 124b->124b; – call to is_even
Block 1250->1252;
If ZF Then
  Block 1263->1267
Else Block 1254->1261 Fi;
Block 1269->1279;
If ZF Then
  Block 1280->1285
Else Block 127b->127b Fi
```

(b) Syntactic Control Flow $f$

**thm:** $MRR \Longrightarrow \{P\}f\{Q;M\}$
**proof:**

```
apply (check_scf_step)+
apply (check_scf_while "P_123e || P_1246")
apply (check_scf_step)+
```

**where:**

$P \equiv \texttt{rip} = 1149 \wedge \texttt{rsp} = \texttt{rsp}_0 \wedge \ldots \wedge *[\texttt{rsp}, 8] = \texttt{ret\_addr}$
$Q \equiv \texttt{rip} = \texttt{ret\_addr} \wedge \texttt{rsp} = \texttt{rsp}_0 + 8 \wedge \ldots \wedge *[\texttt{rsp}_0, 8] = \texttt{ret\_addr}$

(c) Theorem and proof code

$M \quad = \{a = [\texttt{rsp}_0, 8], b = [\texttt{fs}_0 + 40, 8], c = [\texttt{rsi}_0 + 36, 4], d = [\texttt{rsp}_0 - 8, 8], \ldots\}$
$MRR = \{a, b, c, d, \ldots\}$ are separate

(d) The memory regions and their relations for block `123e->1244`.

$$
\begin{aligned}
P_{\texttt{123e}}(\sigma) = \; & \texttt{rip} &&= \texttt{123e}\\
& \texttt{rbp} &&= \texttt{rsp}_0 - 8\\
& \texttt{rdi} &&= \texttt{rdi}_0\\
& \texttt{rsp} &&= \texttt{rsp}_0 - (88 + 16 * ((15 + 4 * \text{sextend}(\langle 31, 0\rangle \texttt{rdi}_0)) \;/\; 16))\\
& *[\texttt{rsp}_0 - 40, 8] &&= \texttt{rsp}_0 - (85 + 16 * ((15 + 4 * \text{sextend}(\langle 31, 0\rangle \texttt{rdi}_0)) \;/\; 16)) >> 2 << 2\\
& *[\texttt{rsp}_0 - 48, 8] &&= \text{sextend}(\langle 31, 0\rangle \texttt{rdi}_0) - 1\\
& *[\texttt{rsp}_0 - 56, 8] &&= \texttt{rsi}_0 + 32\\
& \ldots
\end{aligned}
$$

(e) Invariant at line 0x123e (only 7 out of 23 equations shown)

$$\{P_{\text{124b}}\} \; \boxed{\text{is\_even}} \; \{P_{1250}; M_{is\_even}\}$$

(f) Assumption due to call of function is_even

Fig. 1: An FMUC. Region $[a, s]$ denotes a region of $s$ bytes starting at 64-bit address $a$. Notation $*r$ denotes reading region $r$ in little-endian fashion. Notation $\langle 31, 0\rangle \texttt{rdi}_0$ takes the lower 32 bits of the register.

expresses that the function has returned, i.e., the instruction pointer is equal to the return address and the stack pointer `rsp` is equal to its original value plus eight. For any callee-saved register, i.e., any register whose value is assumed to be preserved by the function call, it will say that its value is unchanged.

The component $f$ of the memory usage theorem is a representation of the control flow of the function in terms of syntactic structures such as basic blocks, loops and if-then-else statements (see Figure 1b). We call this the syntactic control flow (SCF). The SCF is automatically generated from the control flow graph (CFG). The reason that a syntactic structure is required, is because the proof is done using Hoare logic, which is guided by syntax. The proof of an FMUC of an entire function is based on FMUCs per basic block. Thus one FMUC is generated per basic block, and one corollary FMUC for the entire function.

The proof consists of two further proof ingredients: *memory region relations* and *invariants*. We zoom in on block `123e`–>`1244` to explain both of these. The FMUC provides 13 regions for this block, of which 4 are shown (see Figure 1d). Region $a$ stores the return address. Region $b$ depends on the segment register `fs` and stores the canary [15]. Region $c$ is based on the pointer passed as second argument to the function. Finally, region $d$ is part of the stack frame. The generated memory region relations assume that all these regions are separate. Out of the per-block memory regions and their relations, memory regions and relations for the function as a whole are composed.

For each basic block, an *invariant* is generated. Stronger invariants can lead to a tighter approximation of memory usage. The invariant assigned to block `123e`–>`1244` is effectively a loop invariant (see Figure 1e). The frame pointer `rbp` is equal to the original stack pointer minus eight. Register `rdi` has not been touched. We also show some of the more complex invariants, such as the value of the stack pointer. In total, the loop invariant provides information on 11 registers and 12 memory locations for this basic block. Note that the FMUC provides preconditions in terms of the initial state of the corresponding basic block. In Section 3.2 these are lifted to preconditions in terms of the initial state of the function.

For this example, we treated `is_even` as an external function (see Figure 1f). An assumption was thus generated, that expresses that the memory usage of that function suffices to show that the invariant at line `124b` implies the invariant at line `1250`. This means, among others, that the memory used by `is_even` (denoted $M_{is\_even}$) should not overlap with regions $a$ through $d$. Section 3.4 provides more information on composition.

The FMUC is generated automatically, except for the three line proof in Figure 1c. Due to the undecidability of memory usage, interaction may be required. Isabelle/HOL proof strategies are provided to assist in that interaction. Section 3 provides more details. The manual effort required in proving the FMUC for this function, consists simply of calling the proper proof strategies. First, `check_scf_step` is run, applying Hoare logic rules and proving correctness of the memory usage until the loop. Then, the proof strategy for dealing with the loop is called, with the invariant generated from the FMUC. Finally, `check_scf_step` is called again, which is able to verify the remainder of the function.

Finally, note that without any assumptions the function could overwrite its own return address at various places. The memory region relations *MRR* are sufficiently strong to exclude this. These relations thus form the preconditions under which a return-address exploit is impossible. As example, they assume that regions $a$ and $c$ are separate. This means that the address stored in parameter `argv` (reflected as $\texttt{rsi}_0$ at the assembly level) is not allowed to point to a region within the stack frame of function `main`.

Due to space restriction, we omit details on the algorithms that generate an FMUC. In general, none of the FMUC generation is part of the trusted computing base. That is, none of the algorithms need to be backed up by formal proofs. The *output* of the FMUC generation is imported into Isabelle/HOL, where it is proven correct. If there is an error in CFG generation, control flow extraction, symbolic execution, or in the generated invariants, then the certificate cannot be proven in Isabelle/HOL. One exception is the memory region relations. They are assumptions, and if they are internally inconsistent this leads to a vacuous truth. For that reason, Z3 is used to generate them [39], making it impossible to introduce, e.g., a relation where two overlapping regions are considered separate.

## 3 FMUC Verification

This section presents the verification of an FMUC. Both the FMUC and the original assembly are loaded into Isabelle/HOL. The theorem is then proven using the proof ingredients stored in the FMUC. This means that given a step function that models the semantics of the assembly instructions, the Hoare triple is verified.

Let step :: $I \times S \times S \mapsto \mathbb{B}$ be a transition relation. It takes as input an instruction of type $I$ and two states $\sigma$ and $\sigma'$. It returns true if and only if execution of the instruction in state $\sigma$ can produce state $\sigma'$. Undefined behavior, such as null-pointer dereferencing, is modeled by relating a state to any successor state. The semantics of a syntactic control flow (SCF) are straightforwardly defined by a function exec_scf :: $SCF \times S \times S \mapsto \mathbb{B}$ (here $SCF$ denotes the type of a syntactic control flow object). In case of loops the function is defined using a least fixed point construction. This way, if the halting condition is never met, there exists no related $\sigma'$.

First, we define the notion of memory usage wrt. a certain state change:

**Definition 1.** *The set of memory regions $M$ is the* memory usage *wrt. the state change from $\sigma$ to $\sigma'$, if and only if, any byte at an address $a$ not inside one of the regions is unchanged.*

$$\text{usage}(M, \sigma, \sigma') \equiv \forall a \;\cdot\; (\forall r \in M \;\cdot\; [a,1] \bowtie r) \Longrightarrow \sigma' : *[a,1] = \sigma : *[a,1]$$

Here, notation $\sigma : *[a, s]$ means reading in little-endian fashion $s$ bytes from memory address $a$ in state $\sigma$. Notation $r_0 \bowtie r_1$ denotes that two regions are separate.

**Definition 2.** *A memory usage Hoare triple is defined as:*

$$\{P\}\ f\ \{Q; M\} \equiv \forall \sigma\ \sigma'\ \cdot\ P(\sigma) \wedge \text{exec\_scf}(f, \sigma, \sigma') \longrightarrow Q(\sigma') \wedge \text{usage}(M, \sigma, \sigma')$$

In words, Definition 2 states the following: if precondition $P$ holds on the initial state $\sigma$ and $\sigma'$ can be obtained by executing $f$, postcondition $Q$ holds on the produced state and the values stored in all memory regions outside set $M$ are preserved.

### 3.1 Verification Tools Used

**Isabelle/HOL** The theorem prover utilized in this work was Isabelle 2018 [44]. It is a generic tool with a flexible, extensible syntactic framework. Isabelle also utilizes a powerful proof language known as intelligible semi-automated reasoning (Isar) [59] and a proof strategy language called Eisbach [37]. We made heavy use of Word library [17]. This library provides a limited-precision integer type, `'a word`, where `'a` is the number of bits in the integer. Various operations are provided for manipulation of and arithmetic involving formal words, including bit indexing, bit shifting, setting specific bits, and signed and unsigned arithmetic. Operators for inequality are also included, as well as operations for converting between word sizes.

**Machine Model and Instruction Semantics** Heule et al. provide semantics of the x86-64 architecture [27]. Instead of manually codifying instruction semantics, they applied machine learning to derive semantics from a live x86 machine. This produced highly reliable semantics: they compared the semantics to manually written semantics based on the Intel reference manuals, and found that in the few cases where they differed the Intel manuals were wrong. Roessle et al. embedded these semantics into the Isabelle/HOL theorem prover and tested the formal Isabelle semantics against live x86 hardware [49]. This formal machine model is the base of our verification effort.

**Symbolic Execution** Bockenek et al. provide an Isabelle/HOL symbolic execution engine based on the above semantics [6]. Effectively, this provides a function symb_exec that symbolically runs basic blocks. Let $a_0$ and $a_1$ be the start- and end-addresses of the block. A call to symb_exec$(a_0, a_1, \sigma, \sigma')$ returns true if and only if state $\sigma'$ is the result of symbolically executing the block from state $\sigma$. The symbolic execution is completely written in Isabelle/HOL, meaning that every rewrite rule has been formally proven correct.

### 3.2 Per-block Verification

Verification occurs by first verifying per basic block. Figure 2a shows an introduction rule for establishing a Hoare triple over a basic block. The first assumption requires the symbolic execution method to run over a universally quantified symbolic state $\sigma$ that satisfies the precondition. Any resulting state $\sigma'$ should satisfy the postcondition $Q$, and the set of memory regions $M$ generated for the block should be correct.

The second assumption is required because of an important subtlety: the regions generated in the FMUC are expressed in terms of the initial state of their basic block. However, it makes no sense to express the regions used by individual blocks within a larger function in terms of their own initial state. If a region of a basic block somewhere within a function body depends on, e.g., the value of register `rdi` at the start of that block, then it is unsound to express that memory region in terms of $\texttt{rdi}_0$, i.e., the value of `rdi` at the start of the function. Therefore, the Hoare triples are defined based on a set of memory regions $M'$ that solely depends on the initial state of the function. For each block, that set is obtained by taking the generated set of memory regions $M$ (expressed in terms of the initial state of the block) and applying it to any state that satisfies the current invariant. This produces a set of regions expressed in terms of the initial state of the function.

An Isabelle proof strategy has been implemented that, given the proof ingredients from the FMUC, discharges this introduction rule. The proof strategy runs symbolic execution within Isabelle/HOL, proves the postcondition and proves the memory usage. The open variables $P$, $Q$, $a_0$, $a_1$ and $M$ are all provided by the FMUC. No interaction is required; for basic blocks the proof is automated.

### 3.3 Verification of Function Body

$$\frac{\begin{array}{l}\forall \sigma\ \sigma' \cdot P(\sigma) \wedge \text{symb\_exec}(a_0, a_1, \sigma, \sigma') \Longrightarrow Q(\sigma') \wedge \text{usage}(M(\sigma), \sigma, \sigma') \\ M' = \{\ r \mid \exists \sigma \cdot P(\sigma) \wedge r \in M(\sigma)\ \}\end{array}}{\{P\}\ \texttt{Block}\ \texttt{a}_0\texttt{->}\texttt{a}_1\ \{Q; M'\}}$$

(a) Introduction rule

$$\frac{\{P\}\ f\ \{Q; M_1\} \qquad \{Q\}\ g\ \{R; M_2\} \qquad M = M_1 \cup M_2}{\{P\}\ f; g\ \{R; M\}}$$

(b) Sequence rule

$$\frac{\{I \wedge B\}\ f\ \{I'; M\} \qquad I' \Longrightarrow I \qquad I \wedge \neg B \Longrightarrow Q}{\{I\}\ \texttt{While}\ B\ \texttt{DO}\ f\ \texttt{OD}\ \{Q; M\}}$$

(c) While rule

Fig. 2: Hoare rules for memory usage

For each syntactic construct, a Hoare rule is defined (see Figure 2). The sequence and conditional rules (only first is shown) are straightforward: the memory usage is the union of the memory usage of the constituents. Note that the sequence rule is sound only because the memory predicates are independent of the initial state of the basic blocks, as discussed above.

The while rule is based on a loop invariant $I$. If the memory usage of one iteration of function body $f$ is constrained to the set of memory regions $M$, then that holds for the entire loop. This sounds counterintuitive. Consider a simple C-like loop iterating from $i = 0$ while $i < 10$ and as body the assignment $a[i] = 0$, i.e., it writes to the $i$th element of an array. Verification of the loop requires the invariant $I(\sigma) = i(\sigma) < 10$. The FMUC of the loop body will have a set of memory regions $M(\sigma) = \{[a + i(\sigma), 1]\}$, i.e, one region of one byte, expressed in terms of the initial state of the basic block. Now consider the application of the introduction rule to the block of the loop body. It will introduce a Hoare triple with:

$$\begin{aligned} M' &= \{\ r \ \mid\ \exists\sigma\ \cdot\ I(\sigma) \wedge r \in M(\sigma)\} \\ &= \{\ r \ \mid\ \exists\sigma\ \cdot\ i(\sigma) < 10 \wedge r = [a + i(\sigma), 1]\} \\ &= \{\ [a', 1] \ \mid\ a \leq a' \leq a + 10\} \end{aligned}$$

The set $M'$ is actually the memory used by the entire loop. This is because the introduction rule applies the state-dependent set of memory regions to any state that satisfies the invariant. This shows that the strength of the generated invariants influences the tightness of the overapproximation of memory usage. A weaker invariant, e.g., $i < 20$, would produce a larger set of memory regions.

An Isabelle/HOL proof strategy is implemented that automatically applies the proper Hoare logic rule. It is driven by the syntactic control flow provided by the FMUC. For function bodies without loops, this proof strategy requires no further interaction. For each loop entry, it is required to manually apply the weaken rule to show that the postcondition of the block before entry implies the loop invariant. Without exception, each of these proofs could be finished using standard off-the-shelf Isabelle/HOL tools. The part that is usually the most involved – defining the invariants – is taken care of by the FMUC generation.

### 3.4 Composition

Let $f$ be a function body. Assume that the function has been verified, i.e., a Hoare triple has been proven of the form: $\{P_f\}\ f\ \{Q_f; M_f\}$. In order to composably reuse that verification effort, function $f$ is considered to be a black box once it is verified. Now consider a function $g$ calling function $f$:

```
a0: push rbp
a1: call f
a2: pop rbp
a3: ret
```

Let $P$ denote the precondition right before executing the assembly instruction `call`. Precondition $P$ contains the equality $*[\mathtt{rsp}_0^g - 8, 8] = \mathtt{rbp}_0^g$, expressing that function $g$ has pushed frame pointer `rbp` into its own local stack frame. Let $Q$ denote the postcondition just after returning, but before executing `pop`. The postcondition of $g$ expresses that callee-saved register `rbp` is properly restored, i.e., $\mathtt{rbp} = \mathtt{rbp}_0^g$. That is indeed done by the `pop` instruction. In order to prove proper restoration of `rbp`, it must be proven that function $f$ did not overwrite any byte in region $[\mathtt{rsp}_0^g - 8, 8]$. Additionally, function $f$ must be proven not to overwrite region $[\mathtt{rsp}_0^g, 8]$ which stores the return address of $g$. For this particular instance of calling $f$, it thus must be proven that $f$ preserves these two regions.

More generically, function $f$ can be called by various functions other than $g$. For each call the specific requirements on which memory regions are required to be preserved differ. Thus, to be able to verify function $f$ once, and reuse that verification effort for each call, the verification effort must at least contain an overapproximation of the memory written to by function $f$. Note that this is exactly the requirement when using *separation logic* [45,47,33]. Separation logic provides a *frame rule* for compositional reasoning. This frame rule informally states that if a program can be confined to a certain part of a state, properties of this program carry over when the program is part of a bigger system.

We thus provide a version of the frame rule of separation logic, specific to memory usage verification (see Figure 3). Effectively, this rule is used to prove that the memory usage of a caller function $g$ is equal to the memory it uses itself, *plus* the memory used by function $f$. It requires four assumptions. First, it assumes function $f$ has been verified for memory usage, with $M_f$ denoting that memory usage. Second, it assumes that precondition $P$ can be split up into two parts: precondition $P_f$ required to verify function $f$, and a separate part $P_{\mathrm{sep}}$. The separate part is specific to the actual call of the function. In the example, $P_{\mathrm{sep}}$ will contain the equality $[\mathtt{rsp}_0^g - 8, 8] = \mathtt{rbp}_0^g$. Third, the correctness of the set of memory regions $M_f$ should suffice to prove that the separated part $P_{\mathrm{sep}}$ is preserved. In the example, this effectively means that $M_f$ should not overlap with the two regions of $g$. Fourth, $P_{\mathrm{sep}}$ and $Q_f$ should imply postcondition $Q$.

$$\frac{\begin{array}{l}\{P_f\}\ f\ \{Q_f; M_f\}\\ P \Longrightarrow P_f \wedge P_{\mathrm{sep}}\\ \forall \sigma\ \sigma' \ \cdot\ \mathrm{usage}(M_f, \sigma, \sigma') \wedge P_{\mathrm{sep}}(\sigma) \longrightarrow P_{\mathrm{sep}}(\sigma')\\ Q_f \wedge P_{\mathrm{sep}} \Longrightarrow Q\end{array}}{\{P\}\ \mathtt{Call}\ f\ \{Q; M_f\}}$$

Fig. 3: Frame rule for composition of memory usage

In practice, many functions will not be part of the assembly code under verification (e.g., external calls). We thus have to generate the assumptions required to proceed with verification. To this end, we introduce the following notation:

$$\{P\}\ \boxed{\mathrm{f}}\ \{Q; M_f\} \equiv \exists\ P_f\ Q_f\ P_{\mathtt{sep}}\ \cdot\ \text{four assumptions of frame rule are satisfied}$$

Making this assumption informally expresses that function $f$ is assumed to have been verified. Its memory usage $M_f$ is assumed to suffice to prove that we could step from states satisfying $P$ to states satisfying $Q$.

## 4 Case Study: Xen Project

The Xen Project [13] is a mature, widely-used virtual machine monitor (VMM), also known as a *hypervisor*. Hypervisors provide a method of managing multiple virtual instances of operating systems (called guests or domains) on a physical host. The Xen hypervisor is a suitable case study because of its security relevance and its complex build process involving real production code. Security is a significant issue in environments where hypervisors are used, such as the Amazon Elastic Compute Cloud (Amazon EC2), Rackspace Cloud, and many other cloud service providers. For example, when one or more physical hosts support virtual guests for any number of distinct users, ensuring isolation of the guest operating systems (OSs) is important. The Xen build process produces multiple binaries that contain functions not present in the Xen source itself. This is due to the inclusion of external static libraries and programs. We used Xen 4.12 compiled with GCC 8.2 via the standard Xen build process. This build process uses various optimization levels, ranging from `O1` to `O3`.

Of the binaries produced by the Xen build process, we considered three: `xenstore`, `xen-cpuid`, and `qemu-img-xen`. The `xenstore` binary is involved in the functionality of XenStore,[6] a hierarchical data structure shared amongst all Xen domains. The `xen-cpuid` utility queries the underlying processors and displays information about the features they support. The third binary, `qemu-img-xen`, consists of over three hundred functions that are not present in the Xen source code. It provides some of the functionality of Quick Emulator (QEMU). QEMU is a free, open-source emulator.[7] Xen uses it to emulate device models (DMs), which provide an interface for hardware storage.

| Binaries | Function Count | Instruction Count | Loops | Manual Lines of Proof |
|---|---|---|---|---|
| `xenstore` | 2/6 | 100 | 0 | 6 |
| `xen-cpuid` | 2/3 | 210 | 2 | 39 |
| `qemu-img-xen` | 247/343 | 11,942 | 64 | 1,002 |
| Total | 251/352 | 12,252 | 65 | 1,047 |

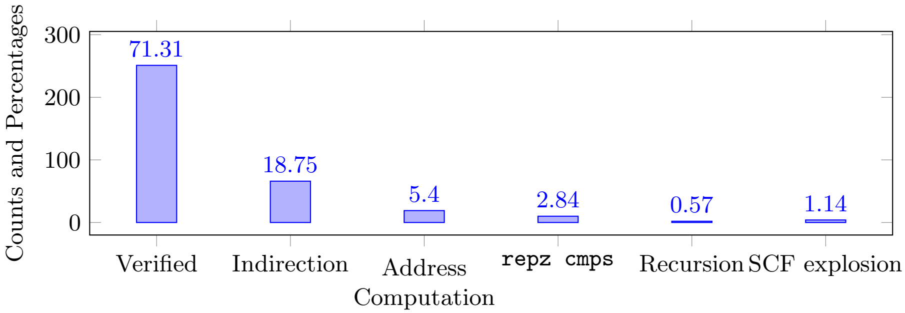

Fig. 4: Case Study Overview

[6] https://wiki.xen.org/wiki/XenStore
[7] https://www.qemu.org/

Our methodology is currently capable of dealing with 71% of the functions present in these binaries (see Figure 4). The supported features include (nested) loops, subcalls, variable argument lists, jumps into other function bodies, string instructions with the `rep` prefix. There is no particular limit on function size. The average number of instructions per function analyzed is 49. Some of the functions analyzed have over 300 instructions and over 100 basic blocks.

There are five categories of features we do not support. The first and most common is *indirection*, accounting for 19%. Indirection involves a call or jump instruction that loads the target address from a register or memory location rather than using a static value. Switch statements and certain uses of `goto` are the most common causes of indirect jumps. Indirect calls generally result from usage of function pointers. For example, the `main` functions of all three verified binaries used switch statements in loops in the process of parsing command line options. These statements introduced indirect branches.

The second category involves issues related to generating the memory region relations. This step requires solving linear arithmetic over symbolically computed addresses. Sometimes, addresses are computed using a combination of arithmetic operators with bitwise logical operators. In some of these cases, our translation to Z3 does not produce an answer. As an example, function `qcow_open` uses the rotate-left function to compute an address. As another example, function `AES_set_encrypt_key` produces addresses that are obtained via combinations of bit-shifting, bit masking, and `xor`-ing.

The instruction `repz cmps` is currently not supported for technical reasons. It is the assembly equivalent of the function `strncmp`, but instead writes its result to a flag. Various other string-related instructions with the `rep` prefix are supported. Functions with *recursion*, a minority in systems code, are also not supported. Recursive stack frames in our framework are not well-suited to automation. The two recursive functions we encountered both perform file-system-like tasks. Functions `do_chmod` and `do_ls` are similar respectively to the permission-setting `chmod` utility, and directory-displaying `ls`. The final category is functions whose SCF explodes. The issue occurs mostly when loops have multiple entries.

The table in Figure 4 provides an overview of the verification effort. The table shows the absolute counts of functions verified as well as the total number of instructions for those functions. Alongside that information is the number of functions with loops that were verified and how many manual lines of proof were required in total. The vast majority of those manual proof lines were related to the loop count.

## 5 Related Work

Assembly verification has been an active research field for decades. Table 1 provides an inexhaustive overview of related work. We first address some formal verification efforts at the assembly level. Then we discuss work in which assembly verification played a role in a larger verification context. Finally, verified compilation and static binary analysis tools are discussed.

**Assembly-level Verification.** Clutterbuck et al. [14] performed formal verification of assembly code using SPACE-8080, a verifiable subset of the Intel 8080 instruction set architecture (ISA) that is analyzable and formally verifiable [12]. Not long after, Bevier et al. presented a systems approach to software verification [5,7]. Their work laid out a methodology for verifying the correctness of all components necessary to execute a program correctly, including compiler, assembler and linker. The methodology was applied to a small OS kernel, Kit [4]. Similarly, Yu and Boyer [60,8] presented operational semantics and mechanized reasoning for approximately 80% of the instructions of the MC68020 microprocessor, over 85 instructions. Their approach utilized symbolic execution of operational semantics. These early efforts required significant interaction. For example, the approach of Yu and Boyer required over 19,000 lines of manually written proof to verify approximately 900 assembly instructions.

Matthews et al. targeted a simple machine model called TINY as well as Java virtual machine (JVM) bitcode using the M5 operational model [38]. Their approach utilizes symbolic execution of code annotated with manually written invariants. It also used verification condition generation to increase automation. This reduced the number of manually written invariants. Both of these assembly-style languages feature a stack for handling scratch variables rather than a register file as x86, ARM, and most other mainstream ISAs do.

Goel et al. presented an approach for modeling and verifying non-deterministic programs on the binary level [25,24]. In addition to formulating the semantics of most user-mode x86 instructions, they provided semantics for common system calls. System call semantics increase the spread of programs that can be fully verified. Their work was applied to multiple small case studies, including a word count program and two kernel-mode memory copying examples.

Bockenek et al. provide an approach to proving memory usage over x86 code [6]. They used a Floyd-style reasoning framework to prove Floyd invariants over functions [21]. They have applied it to functions of the HermitCore unikernel, covering 2,613 assembly instructions. Their approach required a significant amount of manual effort: pre- and postconditions, invariants, the actual regions of memory used and their relations all need to be manually defined.

The main difference between these existing approaches and the methodology presented in this paper concerns automation. Generally, interactive theorem proving over semantics of assembly instructions does not scale due to the amount of intricate user interaction involved. Figure 1e shows, e.g., the complexity of defining an assembly-level invariant even for a small example. Fully automated approaches to formal verification, however, do not scale either. The recent automated approach AUSPICE takes about 6 hours for a 533-instruction string search algorithm [56]. To the best of our knowledge, our methodology is the first that is able to deal with optimized x86-64 binaries produced by production code, with a "manual effort vs. instruction count ratio" of roughly 1 to 11.

Myreen et al. developed *decompilation-into-logic* [40,41,42]. That work, developed in the HOL4 theorem prover [54], uses operational semantics of machine code to lift programs into a functional form. That functional form can then be

Table 1: Overview of Related Work.

| Work | Target | Approach | Applications | Verified code |
|---|---|---|---|---|
| Clutterbuck & Carré | SPACE-8080 | ITP | N/A | |
| Bevier et al. | PDP-11-like | ITP | Kit | |
| Yu & Boyer | MC68020 | ITP | String functions | 863 insts |
| Matthews et al. | Tiny/JVM | ITP+VCG | CBC enc/dec | 631 insts |
| Goel et al. | x86-64 | ITP | word-count | 186 insts |
| Bockenek et al. | x86-64 | ITP | HermitCore | 2,613 insts |
| Tan et al. | ARMv7 | ATP | String search | 983 insts |
| Myreen et al. | ARM/x86 | DiL | seL4 | 9,500 SLoC |
| Feng et al. | MIPS-like | ITP | Example functions | |
| This paper | x86-64 | ITP+CG | Xen | 12,252 insts |
| Sewell et al. | C | TV+DiL | seL4 | 9,500 SLoC |
| Shi et al. | C/ARM9 | ATP+MC | ORIENTAIS | 8,000 SLoC, 60 insts |
| Dam et al. | ARMv7 | ATP+UC | PROSPER | 3,000 insts |

| | | | |
|---|---|---|---|
| VCG | = Verification Condition Generation | DiL | = Decompilation-into-Logic |
| SLoC | = Source Lines of Code | ATP | = Automated Theorem Proving |
| UC | = User Contracts | CG | = Certificate Generation |
| TV | = Translation Validation | MC | = Model Checking |

used in a Hoare logic framework for program analysis [40]. Decompilation-into-logic has been used for both ARM and x86 ISA machine models, and applied to various large examples, including benchmarks such as a garbage collector, and the Skein hash function. Decompilation-into-logic covers – formally – the gap between machine code and a HOL model. It is not a verification method in itself, i.e., it does not verify properties over the machine code. It can be used as a component in a binary-level verification methodology [51].

Feng et al. presented stack abstractions for modular verification of assembly code [20,19]. Their work allows for integration of various proof-carrying code systems [43]. As with our work, it utilizes a Hoare-style framework for its verification. The authors applied their work to multiple example functions, such as two factorial implementations. In constrast to our approach, manual annotations are required to provide information regarding invariants and memory layout.

**Integrated Assembly-Level Verification Efforts.** A major verification effort, based on decompilation-into-logic, is the verification of the seL4 kernel [32,31]. The seL4 project provides a microkernel written in formally proven correct C code. The tool AutoCorres [26] is used for C code verification. Sewell et al. verified a refinement relation between the C source code and an ARM binary for both non-optimized and optimized at `O2` [51]. The major differences with respect to our work is that our methodology targets existing production code, instead of code written with verification in mind. For example, the seL4 source code does not allow taking the addresses of stack variables (such as in Figure 1a): their approach requires a static separation of stack and heap. Neither the seL4 proof effort nor our methodology support function pointers.

Shi et al. formally verified a real-time operating system (RTOS) for automotive use called ORIENTAIS [52]. Part of their approach involved source-level verification using a combination of Hoare logic and abstract communicating sequential processes (CSP) model analysis [29]. Binary verification was done by lifting the RTOS binary to xBIL, a related hardware verification language [53]. They translated requirements from the OSEK automotive industry standard to source code annotations.

Targeting a similar case study as this paper, Dam et al. formally verified a tiny ARMv7 hypervisor, PROSPER [16,3] at the assembly level. Their methodology integrated HOL4 with the Binary Analysis Platform (BAP) [9]. BAP utilizes a custom intermediate language that provides an architecture-agnostic representation of machine instructions and their side effects. HOL4 was used to translate the ARM binary into BAP's intermediate language, using the formal model of the ARM ISA by Fox et al.[22]. The SMT solver Simple Theorem Prover (STP) [23] was used to determine the targets of indirect branches and to discharge the generated verification conditions. While the approach was generally automated, user input was still required to describe software contracts of the hypervisor.

**Verified Compilation.** In contrast to directly verifying machine or assembly code, one can verify source code and then use *verified compilation.* Verified compilation establishes a refinement relation between assembly and source code. The CompCert project [36] provides a compiler for a subset of C. Its output has been verified to have the same semantics as the C source code. The seL4 project used CompCert to reduce its trusted code base [31]. Another example of verified compilation is CakeML [35]. It utilizes a subset of Standard ML modeled with big-step operational semantics. The main purpose of verified compilation, however, is not to verify properties over the code. For example, if the source code is vulnerable to a return-address exploit, then the assembly code is vulnerable as well. Verified compilation is thus often accompanied by source code verification. We have argued that for memory usage, assembly-level verification is necessary.

**Static Analysis.** Static analysis of binary code has been an active research field for decades [34,9,58]. The BitBlaze project [55] provides a tool called Vine which constructs control flow graphs for supplied programs and lifts x86 instructions to its own intermediate language (IL). Though Vine itself is not formally verified, it does support interfacing with the SMT solver STP as well as CVC [1,2]. The tool Infer [10], developed at Facebook, provides in-depth static analysis of LLVM code to detect bugs in C and C++ programs. It utilizes separation logic [47] and bi-abduction [11] to perform its analyses in an automated fashion. It is designed to be integrated into compiler toolchains, in order to provide immediate feedback even in continuous integration scenarios. FindBugs is a static analysis tool for Java code [30]. Rather than relying on formal methods, it uses searches for common code idioms to detect likely bugs. Common errors it highlights include null pointer dereferences, objects that compare equal not having equal hash codes, and inconsistent synchronization. The tool Splint [18] detects buffer overflows and similar potential security flaws in C code. It relies on annotated preconditions to derive postconditions.

The main difference between these static analysis tools and formal verification is that these tools generally are highly suited to find bugs, but are not able to prove absence of them. They generally apply techniques that are formally unsound, such as depth-bounded searches.

## 6 Conclusion

This paper presents an approach to formal verification of memory usage of functions in a compiled program. Memory usage is a property that expresses an overapproximation of the memory used by assembly code. Memory usage is fundamental to compositional verification of assembly code, as compositionality at least requires to prove that functions do not unexpectedly interfere with each others' stack frame. It can also be used to show security-related properties, such as integrity of the return address.

Our approach automatically generates a formal memory usage certificate that includes 1.) a set of memory regions read from and written to, 2.) postconditions that express sanity constraints over the function (e.g., the return address has not been overwritten, callee-saved registers are restored), 3.) proof ingredients such as the preconditions necessary for formal verification. The certificate is loaded into a theorem prover, where it is verified. Since the problem of memory usage is undecidable, we use an interactive theorem prover. The proof ingredients, combined with custom proof strategies, provide a large degree of automation. They deal with memory aliasing, the control flow of the function, and invariants.

The approach is applied to three binaries of the Xen hypervisor. These binaries contain production code and are the result of a complex build chain. They contain, among others, various nested loops, large and compound data structures, variadic functions, and both in- and external function calls. For 71% of the functions of these binaries, a certificate could be generated and verified. For each of these functions, it has at least been formally proven that the return address is not overwritten. The amount of user interaction is roughly 85 lines of proof code per 1,000 lines of assembly code. The greatest bottleneck is in indirect branching, which accounts for 19% of the functions.

In the near future we aim to support indirect branching. This would allow support of switches, callbacks, and pointers to functions. Additionally, we aim to strengthen the invariant generation. Stronger invariants lead to a tighter overapproximation of memory usage. The challenge here is not only to generate these invariants, but to automate their proof as well. Finally, we want to leverage the certificate to target high-level security properties, such as noninterference.

*Data Availability Statement and Acknowledgments* All code and proofs are available in the Zenodo repository: 10.5281/zenodo.3676687. Distribution statement: Approved for public release; distribution is unlimited. This material is based upon work supported by the Defense Advanced Research Projects Agency (DARPA) under Agreement No. HR.00112090028, ONR under grant N00014-17-1-2297, and NAVSEA/NEEC under grant N00174-16-C-0018.
